# Loss or Gain of Lipophilic Bioactive Compounds in Vegetables after Domestic Cooking? Effect of Steaming and Boiling

**DOI:** 10.3390/foods10050960

**Published:** 2021-04-28

**Authors:** Alessandra Fratianni, Annacristina D’Agostino, Serena Niro, Annarita Bufano, Bruno Paura, Gianfranco Panfili

**Affiliations:** Dipartimento di Agricoltura, Ambiente e Alimenti, Università degli Studi del Molise, Via De Sanctis, 86100 Campobasso, Italy; annacristina.dag@gmail.com (A.D.); serena.niro@unimol.it (S.N.); annaritabufano84@gmail.com (A.B.); fobos@unimol.it (B.P.); panfili@unimol.it (G.P.)

**Keywords:** domestic cooking, green leafy vegetables, solid loss, carotenoids, tocols

## Abstract

Lipophilic antioxidants are essential components, which have been pointed as bioactive beneficial for human health. This study aimed at evaluating the effect of domestic cooking (boiling, steaming) on the main carotenoids (lutein and β-carotene) and tocols in four different green leafy vegetables: *Sonchus asper* L. Hill, *Sonchus oleraceus* L., *Spinacia oleracea* L. and *Cichorium intybus* L. The total content of the analyzed compounds was determined following the method of alkaline hydrolysis of the matrix and solvent extraction. The leaching of soluble solids after domestic cooking was found to determine a gain in the investigated bioactive compounds in the cooked vegetables, so to cause an apparent content increase in all leafy vegetables, when expressed as mg/100 g dry matter. Considering solid losses, all lipophilic compounds were not affected by boiling; on the contrary, steaming slightly significantly decreased the contents of lutein and β-carotene (on average 20 and 15%, respectively).

## 1. Introduction

The consumption of vegetables and fruit is associated with different health effects against chronic disorders, due also to their amount of bioactive phytochemicals and micronutrients, such as flavonoids, vitamin C, folates (vitamin B9), tocols, carotenoids and xanthophylls. 

Carotenoids and tocols are an important group of bioactive compounds with an antioxidant activity and health-promoting properties [1,2]. Different carotenoids are precursors of vitamin A and are among the compounds having a great influence on the color of different foods [3,4]. Carotenoids can be present in flowers, fruit and vegetables in their free or ester forms. Lutein, β-cryptoxanthin, zeaxanthin and violaxanthin are the most frequent xanthophylls found as esterified forms in fruits and vegetables [4,5,6]. Different carotenoids, such as α,β-carotene and β-cryptoxanthin, lutein and epoxycarotenoids are provided in the diet by yellow/orange fruits and vegetables, but also by green leafy vegetables [4,5,6,7,8].

Tocols, also known as vitamin E, comprise two groups of vitamers, tocopherols and tocotrienols, occurring in eight forms: α-tocopherol (α-T), β-tocopherol (β-T), γ-tocopherol (γ-T), δ-tocopherol (δ-T) and α-tocotrienol (α-T3), β-tocotrienol (β-T3), γ-tocotrienol (γ-T3) and δ-tocotrienol (δ-T3). Tocols have been demonstrated to prevent certain types of cancer, heart and other chronic diseases [2]. Their main sources are vegetable oils, but they are also found in a large amount in different vegetable products with significant nutritional contents [2,8,9]. 

Most of the green leafy vegetables are consumed in-house after removing no edible parts, washing, cutting and different domestic processes involving boiling, microwave cooking, steaming, stewing and frying. Heat treatments can cause, at different degrees, softening of the tissue, color change, aroma formation and inactivation of compounds considered as anti-nutritional, but also a damage in color, taste and nutritional value. In particular, during boiling, they can produce modifications in cellular structure and composition, the breakdown of food matrix (mainly formed of dietary fiber) that may cause the release in water of compounds with low molecular weight and solid losses [10]. Depending on the processing conditions, the cooking of vegetables can affect their bioactive compound contents, with a consequently significant decrease in nutrients and, therefore, of the nutritional quality [11]. Moreover, the changes in the natural barriers in which some nutrients can be involved may result in the release from the matrix of bioactive components or, for those more polar, their loss in cooking water [11]. Different papers have studied the effect of different domestic cooking procedures on several phytochemicals (polyphenols, carotenoids, tocopherols, glucosinolates) and micronutrients (vitamins and minerals) on vegetables, with somehow contradictory results [11,12,13,14,15,16].

Being lipid soluble, carotenoids and tocols are not significantly lost into water-soluble mediums during processing. However, their content in vegetables can be significantly affected by domestic cooking processes in different ways [11,12,13,14,15]. The interactions between the two factors, vegetables and cooking procedures, were significant [12]. In some cases, a reduction was observed for their thermal lability and their sensitivity to oxidation. The extent of degradation is dependent on temperature, light, oxygen occurrence, pH, water activity and the interactions with other antioxidants [17,18,19,20,21]. Furthermore, the severity and length of heat treatment can induce different carotenoid losses/isomerization [22,23]. These phenomena could also depend on the structure and cellular organization of compounds in the food matrix. Moreover, published papers on fruits or vegetables show an increased stability of carotenoid esters compared to the corresponding not-esterified forms [4,5,6]. In other cases, an increase in compounds was found, and it was attributed to their improved extractability and bioavailability [11,12,13,14,15]. 

In the light of the importance of the in-house cooking treatments in our daily life, the aim of this paper was to a have deeper insight into the effect on the amounts of tocols and carotenoids of two domestic cooking processes, such as conventional boiling and steaming, in four different leafy vegetables. Since solid loss could affect the weight of the resulting cooked vegetables, the influence of the solid loss on the amounts of the analyzed compounds was also investigated.

## 2. Materials and Methods

### 2.1. Plant Material

Four different leafy vegetables, chosen on the basis of their wide diffusion in several traditional recipes of the Mediterranean diet [24], were investigated. They were two wild edible plants, belonging to Sonchus species, *Sonchus asper* L. Hill and *Sonchus oleraceus* L. (Asteraceae), and two more commercial ones, spinach (*Spinacia oleracea* L.) and chicory (*Cichorium intybus* L.). Plants of Sonchus species were collected during the years 2019–2020. The conditions of collection and handling are reported in [8]. *Spinacia oleracea* and *Cichorium intybus* were purchased from local markets. The non-edible portion was discarded; from each sample, a minimum of 500 g of edible portion was gathered and cleaned by removing damaged parts and soil particles. 

### 2.2. Cooking Conditions

Two different cooking methods were tested: conventional boiling and steaming. Cooking conditions were performed by preliminary experiments carried out for each vegetable, considering the minimum cooking time to achieve softness, palatability and taste, according to the consumption habits or to the recipe. Each plant batch was divided into three parts to have at least three repetitions in the experiments. A total of 100 g of leaves was chopped and boiled in a beaker in 1 L of water (1:10 food: water). For conventional boiling, a fresh portion was added to 1 L of boiling water and cooked for 10 min. For steaming, the portion of the vegetable was placed on a steaming rack over boiling water in a closed water bath for 10 min. The boiling water was drained off for 5 min. After cooking and draining, the cooked portions, the water samples and the fresh controls were freeze-dried (Genesis 25SES freeze dryer, VirTis Co., Gardiner, NY, USA), grounded with a refrigerated IKA A10 laboratory mill (IKA®-Werke GmbH & Co. KG, Staufen, Germany), carefully mixed and stored at −20 °C until analysis. The dried water residue was weighed in order to determine the soluble loss after cooking. 

### 2.3. Chemicals and Reagents

Solvents were commercially obtained (Sigma Aldrich, St. Luis, MO, USA), at the highest quality, and used without further purification. All other used reagents were of analytical grade. Lutein was purchased from CaroteNature (Lupsingen, Switzerland); all-trans-β-carotene was from Sigma Chemicals (St. Luis, MO, USA). α-, β-, γ- and δ-Tocopherol standards were from Merck (Darmstadt, Germany); α-, β-, γ- and δ-Tocotrienol standards were purified, as reported in Panfili et al. [25]. Purity for all standards was above 95% (as certified by the suppliers). 

### 2.4. Tocols and Carotenoids Extraction and Quantification

Fresh, freeze-dried plants and water residues were analyzed for moisture, according to the AOAC methods [26]. The procedure for tocols and carotenoids extraction was the saponification method reported in [25,27]. About 0.3 g of milled freeze-dried sample and residue of boiling water was weighed and placed in a screw-capped tube. Then, 5 mL of ethanolic pyrogallol (60 g/L), 3 mL of absolute ethanol, 1 mL of sodium chloride (10 g/L) and 2 mL of potassium hydroxide (600 g/L) for alkaline digestion were added. After nitrogen flushing for 1 min, the tubes were kept for 45 min in a 70 °C water bath and stirred every 5–10 min. After cooling, 15 mL of sodium chloride (10 g/L) were added. Compounds were extracted with 15 mL of n-hexane/ethyl acetate (9:1, *v/v*), until the organic layer was colorless (about three times). Organic layers were collected and evaporated to dryness. Carotenoids were analyzed through the combination of a normal (for xanthophylls) and a reverse phase (for carotenoids) HPLC method. An HPLC Dionex (Dionex, Sunnyvale, CA, USA) analytical system, consisting of a 50 μL injector loop (Rheodyne, Idex Health & Science, Northbrook, IL, USA) and a U6000 pump system was used. For normal phase (NP), samples were suspended in 2 mL of isopropyl alcohol (10%) in n-hexane. The mobile phase was 10% n-hexane: isopropyl alcohol (A) and 20% n-hexane: isopropyl alcohol (B), with the following gradient: 0–6 min (100:0), 16–25 min (50:50), 28–32 min (100:0), respectively, with a flow rate of 1.5 mL/min. The chromatographic separation of the compounds was achieved by means of a 250 × 4.6 mm i.d., 5 µm particle size, 100A Luna Phenomenex Si column (Phenomenex, Torrance, CA, USA) [27]. For the reverse phase (RP), the organic dry residue was dissolved in methanol: MTBE, 50:50 (*v/v*). Separation was performed, at a flow rate of 1 mL/min, by using a 5 μm, C30 YMC (Hampsted, NC, USA) stainless steel column (250 × 4.6 mm i.d.). The mobile phase was methanol: MTBE: water. The gradient profile is given in [28,29]. For tocol analysis, the dry residues were suspended in 2 mL of isopropyl alcohol (1%) in n-hexane, and the analysis was performed through a normal phase HPLC, as already reported for carotenoids and as in [25]. The fluorimetric detection of all tocols was performed by means of a Dionex RF 2000 spectrofluorimeter, at an excitation wavelength of 290 nm and an emission wavelength of 330 nm. Carotenoids were spectrophotometrically detected at 450 nm. Standards were spectrophotometrically quantified and identified through their spectral characteristic. Compounds were identified by comparison of their retention times and Uv/Vis spectra with known commercially available standard solutions. 

### 2.5. Statistical Analysis

The samples were analyzed in triplicate. The results were reported as the average of three determinations. The ANOVA test was applied to data, by using a Statistical Software Package for Windows (SPSS Inc., Chicago, IL, USA). Significance of difference was defined at *p* < 0.05.

## 3. Results and Discussion

The amounts of the single carotenoids, expressed as mg/100 g dry matter (d.m.), in fresh and cooked samples, at the end of each domestic boiling and steaming treatment, for every investigated vegetable, are reported in Table 1. Results are expressed on a dry matter basis (d.m.), to allow a good comparison, taking into account possible moisture change. 

In all analyzed leafy vegetables, lutein and β-carotene were the main detected carotenoids, and they are the only ones discussed in this paper. Data on the other carotenoids are, therefore, not reported. In fresh samples, lutein ranged from about 60 mg/100 g d.m. in Sonchus species to about 100 mg/100 g d.m. in *Sp. oleracea*. Beta-carotene went from about 20 mg/100 g d.m. in Sonchus species to about 60 mg/100 g d.m. in *C. intybus*. These data were confirmed by literature studies on Sonchus species [8,30] and other different green leafy vegetables [4,31]. Among tocols, only α-tocopherol (α-T), from about 20 mg/100 g d.m. in Sonchus species to about 33 mg/100 g d.m. in the others, and γ-tocopherol (γ-T), from about 3 to 14 mg/100 g d.m. in *C. intybus*, were detected. No tocotrienols were found. References for tocols in the investigated species are in accordance with those reported by different authors, for Sonchus [8,30] and for other green vegetables [32,33,34].

A general significant increase (mg/100 g d.m.) in all compounds as to fresh vegetables was found after the boiling treatment (Table 1). In particular, the increase in carotenoids ranged up to 50% for lutein (*S. oleraceus*), to about 65% for β-carotene (*S. asper*). A similar trend was observed for tocols, with increments of α-T going from 25% (*Sp. oleracea*) to 60% (*S. oleraceus*) and of γ-T ranging from about 12% (*C. intybus*) to about 75% (*S. asper*). Results on the effects of domestic cooking on the investigated liposoluble compounds are controversial [11,12,13,14,15,33,34]. Some authors reported losses of carotenoids and tocols after cooking, some others did not observe significant changes, some concluded that thermal processing increases compound concentrations. Regarding carotenoids, when lower temperatures were applied, they seemed more stable during water cooking. Boiling was reported by some authors to be the most destructive water cooking process, while steaming the least [13]. In different cases, particularly after water cooking, increased concentrations in comparison with the fresh uncooked samples were reported. This phenomenon was explained to be due to the enhanced carotenoid chemical extractability from the plant tissue after heating, following disintegration of the plant matrix, cellular breakage and dissociation of molecular linkages between food components, such as carotenoid–protein complexes of the chloroplasts [11,12,13,14,15]. The overall results are variable, since the compounds behavior during cooking could depend on the part of the cooked vegetable, the particle size of the vegetable, its shape and tissue structure and, as a consequence, on the plant under investigation [12,13,14]. There are relatively few studies on the processing, storage and cooking effects on vitamin E in fruits and vegetables, with controversial results, depending on the type of food and cooking time [11,32,33,34]. As already observed for carotenoids, some of these studies found, on average, higher levels of α-tocopherol than in the fresh products, which was suggested to be due to the increment of the chemical extractability of lipidic molecules through heat treatment [11,33,34]. Moreover, greater extractability has been usually associated to a greater bioavailability [35,36], even though, in most cases, this hypothesis was not assessed through proper methods of investigation [36].

It is worth noticing that, in almost all papers, the observed greater extractability from the food matrix refers to free compounds determined after a solvent extraction method. In several cases, the extraction phase is followed by a saponification of the extract, in order to hydrolyze esters or to remove compounds that could interfere with the chromatographic analysis. The statement “greater extractability” after the cooking treatments implies that the extraction method used by the authors was not able to correctly quantify the contents of the investigated compounds, in an untreated matrix. Few papers use a method involving an alkaline hydrolysis (saponification) of the food matrix followed by solvent extraction, which, allowing the opening up of the cell wall matrix and releasing compounds that might be strongly linked to cellular components, can cause a more effective and complete extraction of compounds, thus giving more reliable and comparable results. By applying the cited method, not only free compounds or those de-esterified by saponification, but also compounds difficult to access to the solvent or linked to the food matrix are extracted. We have already proven this procedure for its reliability, and it has been successfully used for cereals and other vegetable samples [4,5,8,17,19,25,27]. The same method was applied in this paper for the extraction of lipophilic compounds from fresh and processed vegetables. For this reason, the observed increases reported in Table 1 after cooking could not be ascribed to a greater extractability from the plant tissue caused by thermal treatments.

As already pointed out, the breakdown of the food matrix and the release of compounds, due to thermal treatments, can cause weight changes due to solid loss. Therefore, apart from moisture loss or gain, solid loss might also be taken into consideration [37]. Regarding the influence of solid losses, in an old paper by Baloch et al. [38], the incomplete extraction of pigments from raw vegetables and/or leaching of soluble solids during processing of the vegetables were considered as the possible explanations for the apparent increase in carotenoid content during processing. In fact, values were found to increment only if they were expressed on dry weight basis. The latest evidence was also discussed in a recent paper by Diamante et al., in colored cauliflowers [33]. The soluble solid losses in water from 100 g lyophilized samples after cooking are reported in Table 2.

Solid losses went from 21% in *Sp. oleracea* to 36% in *S. oleraceus*. As expected, for steamed vegetables, they were to a less extent (about 5 and 10% for *C. intybus*). 

The content of carotenoids and tocols of Table 1 was, therefore, corrected, either in processed foods, or in the cooking water, considering the solid loss, as reported in Table 3 (*S. oleraceus*), Table 4 (*S. asper*), Table 5 (*Sp. oleracea*) and Table 6 (*C. intybus*).

The lipophilic property of the analyzed compounds limited their leaching in water, so that only 1% or less of their initial total amounts were found in the boiling water. No detectable amounts were found in the steaming water. By summing the contents in the residues in water resulting from boiling with those in the cooked samples, no significant differences in the investigated carotenoid and tocol amounts were found, so to conclude that boiling did not affect the measured compounds. The initially found increments of carotenoids and tocols in the cooked samples (Table 1) were, therefore, consequences of the leaching in the cooking water of the solids initially present in the raw samples. This loss determined a gain in bioactive compounds in the cooked vegetables, when data were expressed as mg/100 g d.m. 

A rather small, but significant, decrease due to steaming was observed for lutein (on average 20%) and β-carotene (on average 15%) in all vegetables, with the exception of β-carotene of *Sp. oleracea*. A more marked stability was found for tocols. 

The extent of the degradation of the investigated compounds could depend on temperature, available oxygen and oxidative enzymes. Since no effect was observed due to the boiling temperature, the same could be stated for steaming, where the temperature range is in the same order of magnitude (97–100 °C) [37]. During the steaming treatment, the adopted process conditions could not have been effective in having an enzymatic inactivation; therefore, the activities of different oxidative enzymes, such as peroxidase and lipoxygenase, may have influenced the levels of the analyzed carotenoids in the early stages of the process [39,40].

As already stated, it is difficult to assess from the literature a general effect of food processing. Different domestic processes have proven to have different impacts on carotenoids and tocols, due to their stability upon heating time and temperature, exposure to oxygen and light and the matrix in which they are involved. Data emerging from this work confirm the hypothesis that leaching of soluble solids could be the cause of the apparent increases in boiled samples. Apart from the paper by Baloch et al., 1977 [38], to our knowledge, up to now, there are no experimental papers demonstrating soluble leaching as one of the causes of the observed apparent increase in liposoluble compounds after the boiling treatment.

## 4. Conclusions 

Data emerging from this research demonstrate that, in order to have reliable data of the effect on in-house processing on liposoluble pigments, a complete extraction procedure, together with the evaluation of the solid loss, ought to be taken into consideration. In fact, under our experimental conditions, the use of the saponification procedure of the matrix followed by solvent extraction allowed a complete extraction of total tocols and carotenoids from the investigated vegetable samples. Therefore, the initially observed increments of bioactives after the applied in-house treatments could not be related to their higher extractability, due to the breakdown of the food matrix by the high temperatures. Leaching of soluble solids during processing could, therefore, be the cause of the observed increased amounts in boiled vegetables, when data are expressed as mg/100 g d.m. By considering solid losses, boiling did not significantly affect the main carotenoids and tocols in the investigated vegetables, while steaming had a small effect on their amounts. Further experiments are needed in order to investigate the nature of the solids solubilized in the cooking water, also in relation to different vegetables, with a different tissue structure. Finally, since the thermal lability of carotenoids could also be influenced by their chemical structure, the behavior of the single found compound, as a result of thermal treatments, should be evaluated.

## Figures and Tables

**Table 1:** Contents of the main carotenoids and tocols in the investigated leafy vegetables before and after cooking (mg/100 g d.m.).

Carotenoids	Treatment	*S. oleraceus*	*S. asper*	*Sp. oleracea*	*C. intybus*
Lutein	fresh	57.3 ^a^	60.5 ^a^	109.1 ^a^	87.1 ^a^
	boiling	86.7 ^b^	83.7 ^b^	122.8 ^b^	115.2 ^b^
	steaming	51.4 ^a^	47.9 ^c^	91.8 ^c^	84.9 ^a^
β-Carotene	fresh	17.7 ^a^	22.2 ^a^	39.6 ^a^	56.6 ^a^
	boiling	25.7 ^b^	36.9 ^b^	44.9 ^b^	82.8 ^b^
	steaming	12.4 ^a^	18.2 ^c^	40.9 ^b^	55.4 ^a^
Tocols					
α-T	fresh	19.7 ^a^	20.5 ^a^	32.3 ^a^	33.1 ^a^
	boiling	31.7 ^b^	27.4 ^b^	41.0 ^b^	43.3 ^b^
	steaming	22.6 ^a^	18.1 ^a^	31.3 ^a^	39.6 ^a^
γ-T	fresh	3.3 ^a^	2.7 ^a^	4.7 ^a^	14.1 ^a^
	boiling	5.3 ^b^	4.7 ^b^	6.4 ^b^	15.8 ^b^
	steaming	4.1 ^a^	3.9 ^c^	4.7 ^a^	10.9 ^c^

For each compound, different letters in the same column indicate a statistically significant difference at *p* < 0.05.

**Table 2:** Soluble solid losses (g) in cooking water from 100 g of dry matter of the cooked samples.

Vegetables	Fresh	Cooking Water	
		Boiling	Steaming
*S. oleraceus*	100	36	5
*S. asper*	100	35	5
*Sp. oleracea*	100	21	4
*C. intybus*	100	23	10

**Table 3:** Contents of the main carotenoids and tocols in fresh vegetables (mg/100 g d.m.), in cooked samples (mg/g d.m.) and in cooking water (mg/g d.m.) of *S. oleraceus.*

	Fresh	Boiling			Steaming		
Compounds	100 g	Cooked (A) (64 g) *	Water (B) (36 g) *	A + B (100 g) ^§^	Cooked (C) (95 g) *	Water (D) (5 g) *	C + D (100 g) ^§^
Carotenoids							
Lutein	57.3 ^a^	55.5	0.5	56.0 ^a^	48.8	n.d.	48.8 ^b^
β-Carotene	17.7 ^a^	16.5	0.1	16.6 ^a^	11.9	n.d.	11.9 ^b^
Tocols							
α-T	19.7 ^a^	20.3	0.1	20.4 ^a^	21.4	n.d.	21.4 ^a^
γ-T	3.3 ^a^	3.4	n.d.	3.4 ^a^	3.9	n.d.	3.9 ^a^

* Partition of 100 g of the dry matter of raw samples in cooked samples and in cooking water. ^§^ Sum of the contents in cooked samples and in cooking water (mg/100 g d.m.) Different letters in the same row indicate a statistically significant difference at *p* < 0.05. n.d.: not detectable.

**Table 4:** Contents of the main carotenoids and tocols in fresh vegetables (mg/100 g d.m.), in cooked samples (mg/g d.m.) and in cooking water (mg/g d.m.) of *S. asper*.

	Fresh	Boiling			Steaming		
Compounds	100 g	Cooked (A) (65 g) *	Water (B) (35 g) *	A + B (100 g) ^§^	Cooked (C) (95 g) *	Water (D) (5 g) *	C + D (100 g) ^§^
Carotenoids							
Lutein	60.5 ^a^	54.4	0.5	54.9 ^a^	45.5	n.d.	45.5 ^b^
β-Carotene	22.2 ^a^	24.0	1.7	25.7 ^a^	17.3	n.d.	17.3 ^b^
Tocols							
α-T	20.5 ^a^	17.8	0.2	18.0 ^a^	17.2	n.d.	17.2 ^a^
γ-T	2.6 ^a^	3.1	n.d.	3.1 ^a^	3.7	n.d.	3.7 ^a^

* Partition of 100 g of the dry matter of raw samples in cooked samples and in cooking water. ^§^ Sum of the contents in cooked samples and in cooking water (mg/100 g d.m.). Different letters in the same row indicate a statistically significant difference at *p* < 0.05. n.d.: not detectable.

**Table 5:** Content of the main carotenoids and tocols in fresh vegetables (mg/100 g d.m.), in cooked samples (mg/g d.m.) and in cooking water (mg/g d.m.) of *Sp. oleracea.*

	Fresh	Boiling			Steaming		
Compounds	100 g	Cooked (A) (79 g) *	Water (B) (21 g) *	A + B (100 g) ^§^	Cooked (C) (96 g) *	Water (D) (4 g) *	C + D (100 g) ^§^
Carotenoids							
Lutein	109.1 ^a^	97.0	2.1	99.1 ^a^	88.2	n.d.	88.2 ^b^
β-Carotene	39.6 ^a^	35.5	1.7	37.2 ^a^	39.3	n.d.	39.3 ^a^
Tocols							
α-T	32.3 ^a^	32.4	0.2	32.6 ^a^	30.0	n.d.	30.0 ^a^
γ-T	4.7 ^a^	5.0	n.d.	5.0 ^a^	4.5	n.d.	4.5 ^a^

* Partition of 100 g of the dry matter of raw samples in cooked samples and in cooking water. ^§^ Sum of the contents in cooked samples and in cooking water (mg/100 g d.m.). Different letters in the same row indicate a statistically significant difference at *p* < 0.05. n.d.: not detectable.

**Table 6:** Content of the main carotenoids and tocols in fresh vegetables (mg/100 g d.m.), in cooked samples (mg/g d.m.) and in cooking water (mg/g d.m.) of *C. intybus.*

	Fresh	Boiling			Steaming		
Compounds	100 g	Cooked (A) (77 g) *	Water (B) (23 g) *	A + B (100 g) ^§^	Cooked (C) (90 g) *	Water (D) (10 g) *	C + D (100 g) ^§^
Carotenoids							
Lutein	87.1 ^a^	88.7	0.7	89.4 ^a^	76.4	n.d.	76.4 ^b^
β-Carotene	56.6 ^a^	63.8	0.6	64.4 ^a^	49.9	n.d.	49.9 ^b^
Tocols							
α-T	33.1 ^a^	33.3	0.2	33.5 ^a^	35.5	n.d.	35.5 ^a^
γ-T	14.1 ^a^	12.2	n.d.	12.2 ^a^	9.9	n.d.	9.9 ^b^

* Partition of 100 g of the dry matter of raw samples in cooked samples and in cooking water. ^§^ Sum of the contents in cooked samples and in cooking water (mg/100 g d.m.). Different letters in the same row indicate a statistically significant difference at *p <* 0.05. n.d.: not detectable.

## Data Availability

The data presented in this study are available on request from the corresponding author.
